# Investigation of Cross-Language and Stimulus-Dependent Effects on the McGurk Effect with Finnish and Japanese Speakers and Listeners

**DOI:** 10.3390/brainsci13081198

**Published:** 2023-08-13

**Authors:** Kaisa Tiippana, Yuta Ujiie, Tarja Peromaa, Kohske Takahashi

**Affiliations:** 1 Department of Psychology and Logopedics, University of Helsinki, 00014 Helsinki, Finland; 2 Department of Psychology, College of Contemporary Psychology, Rikkyo University, Saitama 352-8558, Japan; 3 Research Organization of Open Innovation and Collaboration, Ritsumeikan University, Osaka 567-8570, Japan; 4 College of Comprehensive Psychology, Ritsumeikan University, Osaka 567-8570, Japan

**Keywords:** audiovisual, cross-language, Finnish, Japanese, McGurk effect, speech perception, stimulus features

## Abstract

In the McGurk effect, perception of a spoken consonant is altered when an auditory (A) syllable is presented with an incongruent visual (V) syllable (e.g., A/pa/V/ka/ is often heard as /ka/ or /ta/). The McGurk effect provides a measure for visual influence on speech perception, becoming stronger the lower the proportion of auditory correct responses. Cross-language effects are studied to understand processing differences between one’s own and foreign languages. Regarding the McGurk effect, it has sometimes been found to be stronger with foreign speakers. However, other studies have shown the opposite, or no difference between languages. Most studies have compared English with other languages. We investigated cross-language effects with native Finnish and Japanese speakers and listeners. Both groups of listeners had 49 participants. The stimuli (/ka/, /pa/, /ta/) were uttered by two female and male Finnish and Japanese speakers and presented in A, V and AV modality, including a McGurk stimulus A/pa/V/ka/. The McGurk effect was stronger with Japanese stimuli in both groups. Differences in speech perception were prominent between individual speakers but less so between native languages. Unisensory perception correlated with McGurk perception. These findings suggest that stimulus-dependent features contribute to the McGurk effect. This may have a stronger influence on syllable perception than cross-language factors.

## 1. Introduction

The McGurk effect is an illusion in which viewing incongruent visual articulation alters the auditory perception of a speech syllable. The classic example of a McGurk stimulus is auditory /ba/ presented simultaneously with a visual /ga/, i.e., A/ba/Vga/ [1]. Even when the auditory /ba/ is heard correctly when presented alone, perception changes when the McGurk stimulus is presented, so that often /da/ or /ga/ is heard.

Many researchers consider only /da/ responses as an index of the strength of the McGurk effect, because then the participant perceives neither the A nor V component but their fusion [2,3]. In contrast, several researchers interpret all responses other than auditory correct ones as the McGurk effect, indicating a stronger illusion the fewer auditory correct responses are given [4,5,6]. In this view, the McGurk effect arises also when the participant hears the consonant according to the V component. Crucially, despite these differences in interpretation, the McGurk effect shows that seeing the talking face can change the auditory speech percept categorically.

The McGurk effect has had a strong impact on speech research, because it shows that visual speech can also influence speech perception when auditory processing is intact (e.g., no hearing impairment or noisy conditions). It has been used as an index of visual influence on speech perception in hundreds of studies. It has been demonstrated in many languages (e.g., [7,8,9,10]). For excellent reviews with a thorough treatment of the impact and interpretation of the McGurk effect, see [11,12].

One topic that has been explored using the McGurk effect as a tool is cross-language speech perception. It is important to understand the factors influencing language processing in one’s mother tongue and foreign languages, and the contribution of visual speech articulation is a factor in understanding spoken language [8]. Knowledge of how familiar or foreign talking faces and voices influence speech perception may be useful to enhance speech understanding and maybe even language learning [13,14].

Many studies have investigated cross-language effects in audiovisual speech perception, but the findings are mixed. Some studies have found that visual speech has a stronger influence when the listener’s mother tongue, i.e., first language (L1), is different from the speaker’s L1 [15,16]. A seminal study by Sekiyama and Tohkura (1993) showed an increased McGurk effect for Japanese listeners when the speaker was American-English-speaking and correspondingly for American English listeners when the speaker was Japanese-speaking [15]. They proposed that this foreign language effect may arise when the acoustic features deviate from those learned with one’s mother tongue, and consequently, the listener relies more on visual features, resulting in an enhanced McGurk effect for a foreign language speaker.

However, the opposite has also been reported, i.e., the McGurk effect has been reported to be stronger when the speaker has the same L1 as the listener compared with a foreign speaker [17]. Recently, Ujiie and Takahashi (2022) found that Japanese listeners gave more /ta/ (and correspondingly fewer /pa/) responses to stimulus A/pa/V/ka/ when the speaker was Japanese-speaking than when they were an American English speaker [17]. Still, other researchers have reported no difference in the McGurk effect between two different L1s [18], or the findings have been mixed [19,20]. For an example of the latter, Chen and Hazan (2009) found a foreign language effect in Chinese participants with English stimuli but not vice versa [19].

There are several potential sources for these mixed findings. Many different languages have been used in cross-language studies of the McGurk effect. It is possible that differences in phoneme inventories may affect cross-language effects, even when the studied speech sounds are native in the relevant languages [19]. Also, very few speakers and listeners were used in most studies, even though it is now known that the variability between both speech stimuli and individual participants is large, necessitating several stimuli and large sample sizes to obtain reliable results regarding cross-language comparisons [18,21]. Also, the majority of studies have compared English with other languages. English is the modern lingua franca to which people are at least exposed globally, and it is extremely popular to study as a foreign language. Bilinguals experience the McGurk effect more than monolinguals [22]. Thus, the familiarity or knowledge of English may confound foreign language influences, since often the other-language participants know at least some English; meanwhile, native English participants are monolingual.

Our aim was to study potential cross-language effects in Finnish (FI) and Japanese (JP) participants with Finnish and Japanese speakers. Finnish is a Finno-Ugric language in Northern Europe, and Japanese is a Japonic language in East Asia. An advantage of these languages is that neither participant group knew the other language and had been minimally exposed to it. Consequently, the confounding factor of knowledge of the other language was eliminated.

We used the unvoiced counterpart of the classical McGurk stimulus, A/pa/V/ka/, as well as three plosive consonant syllables, /ka/, /pa/ and /ta/ in unisensory and congruent audiovisual presentations, because these unvoiced plosives are native consonants in both Finnish and Japanese. 

The syllables were uttered by eight different speakers: two FI females, two FI males, two JP females and two JP males. Consequently, cross-language comparisons were based on the results obtained from four different speakers in each language. Furthermore, each speaker provided five articulations of each syllable, which reduced the risk of very stimulus-specific cues defining the categorization (e.g., avoiding the event that an exceptionally clear pursing of the lips for V/ba/ in a single video could lead to overestimation of visual speech recognition accuracy). To obtain reliable individual estimates of the McGurk effect, each participant was presented with 50 McGurk trials per speaker.

Our main research question concerned the cross-language effect. If there is a foreign language effect, Finnish participants should give fewer Pa responses to JP than FI McGurk stimuli. And conversely, Japanese participants should give fewer Pa responses to FI than JP McGurk stimuli. The accompanying changes in Ka and Ta responses were also analyzed to determine the type of visual influence in the McGurk effect. The results were analyzed using logistic regression analyses with generalized linear mixed model, taking into account potential differences in stimuli to extract cross-language effects.

In addition to cross-language effects, we wanted to look at differences between individual speakers. The incidence of the McGurk effect varies between stimuli/speakers [18]. It is still unclear which factors contribute to these differences. One factor is unisensory intelligibility.

The McGurk effect is influenced by the unisensory components of the stimulus [23,24]. Regarding visual plosive consonants, velars /g, k/ are often confused with alveolars /d, t/, because the visible articulations look very similar on a talking face; the place of articulation being inside the mouth [23,25]. It is important to determine how this confusability contributes to the fusion effect for a McGurk stimulus. Also, if the auditory component is not recognized perfectly, its confusion pattern is reflected in the response distribution to the McGurk stimulus [23]. We added white noise to the auditory stimuli (at a signal-to-noise ratio (SNR) of 0 dB) to decrease A reliability and to emphasize visual influence in AV conditions. We tested whether responses to A and V speech correlated with those to the McGurk stimulus to address the role of unisensory perception in the McGurk effect.

Previously, it has been found that correct recognition of the place of articulation correlates with McGurk fusions [26,27]. However, if visual perception is directly reflected in the McGurk effect, and a visual velar is correctly recognized, a velar response could also be expected to the McGurk stimulus. On the other hand, if a visual velar is confused with an alveolar, a McGurk “fusion” response could be expected. In the current study, this would mean a positive correlation between visually correct Ka responses to V/ka/ and Ka responses to the McGurk stimulus A/pa/V/ka/ and a positive correlation between Ta (confusion with alveolar) responses to V/ka/ and Ta (“fusion”) responses to the McGurk stimulus. In addition, if more reliable auditory perception is linked with a weaker McGurk effect, there should be a positive correlation between correct Pa responses to A/pa/ and Pa responses to the McGurk stimulus.

In sum, we investigated cross-language effects in audiovisual speech perception with Finnish and Japanese participants and stimuli. Furthermore, we studied how the unisensory components spoken by different speakers influenced the McGurk effect. In Experiment 1, Finnish participants categorized auditory, visual and audiovisual consonant–vowel syllables uttered by Finnish and Japanese speakers. In Experiment 2, Japanese participants conducted the same experiment.

## 2. Materials and Methods

### 2.1. Participants

In Experiment 1, there were 49 Finnish participants (35 females, 13 males and 1 undisclosed) who took part in the experiment. They were native-Finnish-speaking adults (mean age 22 years, range 18–36) without any hearing, vision or neurological problems. Among them, 46 were right-handed. None of them had learned Japanese, nor had any experiences interacting with Japanese people in everyday situations. The University of Helsinki Ethical Review Board in Humanities and Social and Behavioral Sciences has reviewed the study and stated that it is ethically acceptable.

In Experiment 2, 50 Japanese participants (30 females and 20 males) took part in the experiment. They were native-Japanese-speaking adults without any hearing, vision or neurological problems (mean age 21 years; range 18–24). Among them, 46 were right-handed. None of them had learned Finnish nor had any experiences interacting with Finnish people in everyday situations. One participant (female) was excluded from the following analyses because of not completing all the tasks. Ethical approval was obtained from the Research Ethics Review Board of Ritsumeikan University.

### 2.2. Stimuli and Equipment

**Figure 1:**
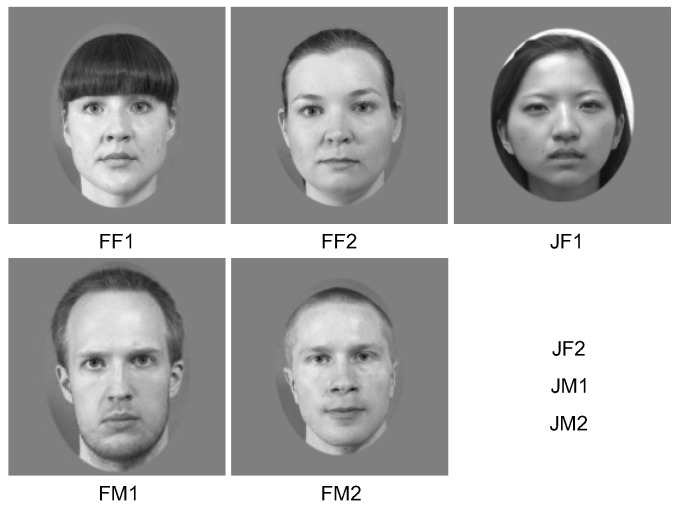
Example frames of stimulus videos. Five out of eight speakers gave permission to publish their pictures: FF1 = Finnish female 1, FF2 = Finnish female 2, FM1 = Finnish male 1, FM2 = Finnish male 2, JF1 = Japanese female 1, JF2 = Japanese female 2, JM1 = Japanese male 1, JM2 = Japanese male 2.

Stimuli were audio and video recordings of syllables /pa/, /ka/ and /ta/, each spoken five times (giving five samples per syllable) by eight speakers: four native Finnish speakers, two females and two males, and four native Japanese speakers, two females and two males (Figure 1).

The auditory stimuli were spoken syllables equalized in intensity. They were presented at a 52 dB(A) sound level with added white noise to produce an SNR of 0 dB. The average duration was 222 ms for FI and 316 ms for JP syllables.

The visual stimuli were videos of the speakers’ faces articulating syllables. They were converted into greyscale and presented with oval windowing, which had a medium grey background. Face width was 6 deg. The video duration was 1000 ms. 

For the audiovisual stimuli, the above mentioned auditory and visual stimuli were presented in synchrony with audio onset (start of the consonant burst) 400 ms after the beginning of the video, preserving the original timing. In the congruent AV stimuli, the auditory stimulus corresponded to the visual stimulus of the original video recording, i.e., the voice and face said the same syllable. In the McGurk stimulus, the auditory and visual stimuli were incongruent, i.e., auditory /pa/ was presented with visual /ka/ (stimulus A/pa/V/ka/). In all McGurk stimuli, the onset of the burst of A/pa/ was aligned with the original onset of the burst of A/ka/. Twenty-five instances of the McGurk stimulus were created for each speaker by combining each of the five A/pa/ samples with each of the five V/ka/ samples.

The experiments were run in Matlab environment (Version R2017b, Mathworks, Inc., Natick, MA, USA) using Psychtoolbox extensions (Version 3.0.141) on a standard PC (Windows 10). Visual stimuli were presented on a 24.1-inch monitor (Eizo CG247 LCD in Exp. 1 and Eizo CS2420 CRT in Exp. 2) with 60 Hz refresh rate. Sound stimuli were delivered via headphones (Beyerdynamic DT 770 Pro in Exp. 1 and Yamaha HPH-200 in Exp. 2).

### 2.3. Procedure

Auditory, visual and audiovisual stimuli were presented in separate blocks. Each stimulus sample was presented once in a block in random order. Thus, A and V blocks had 120 trials (3 syllables × 8 speakers × 5 samples). AV blocks consisted of congruent and McGurk stimuli and had 320 trials (3 congruent syllables × 8 speakers × 5 samples + 25 incongruent syllables × 8 speakers). 

The blocks were run in the following order in Exp. 1: A, V, AV, A, V, A, V, AV, A, V. The duration of the entire experimental session was about 1 ½ hours in Exp. 1. To reduce the duration to about one hour, fewer unisensory blocks were run in Exp. 2 in the following order: A, V, AV, A, V, AV. Thus, in both experiments, each congruent AV syllable was presented 10 times and the McGurk stimulus 50 times per speaker, since the McGurk stimulus was of main interest. Each unisensory syllable was presented 20 times in Exp. 1 and 10 times in Exp. 2 per speaker.

The participants responded after each trial by pressing the appropriate key, labeled Ka, Pa or Ta, according to what they heard the speaker say in the A and AV blocks and according to what they saw the speaker say in the V blocks. They were not explicitly told about the language background or number of speakers. There was also the response option “other” in Exp. 2.

### 2.4. Data Analyses

We analyzed the data using R (version 3.6.1., by RStudio version 1.2.1335 for Windows, RStudio Team, PBC, Boston, MA, USA). The R library “lme4” was used in analyses. We treated data as binomial since participants’ responses frequently violate the assumption of standard analyses of variance that the data are normally distributed and have equal variances [28]. We conducted logistic regression analyses with a generalized linear mixed model (GLMM).

## 3. Results

### 3.1. The McGurk Effect

**Figure 2:**
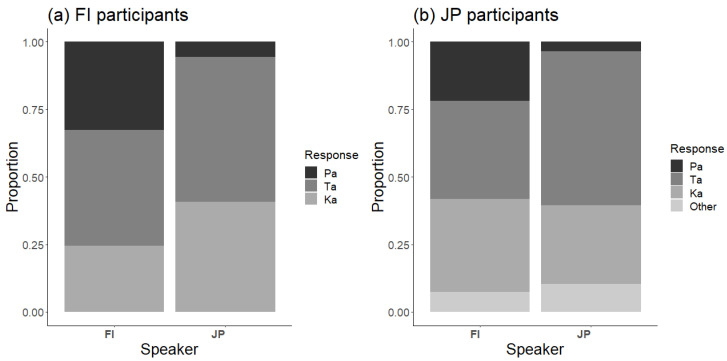
Response distributions to the McGurk stimulus (A/pa/V/ka/) for the Finnish and Japanese speakers in (**a**) Finnish and (**b**) Japanese participants.

The response distributions to the McGurk stimulus with all Finnish and Japanese speakers and participants are shown in Figure 2. To a large extent, the response patterns appear rather similar, with few Pa responses and many Ta and Ka responses, indicating a strong McGurk effect.

**Figure 3:**
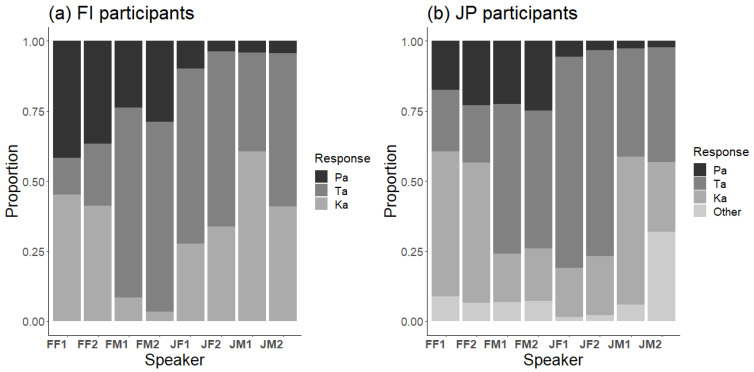
Response distributions to the McGurk stimulus (A/pa/V/ka/) separately for each of the eight speakers in (**a**) Finnish and (**b**) Japanese participants: FF1 = Finnish female 1, FF2 = Finnish female 2, FM1 = Finnish male 1, FM2 = Finnish male 2, JF1 = Japanese female 1, JF2 = Japanese female 2, JM1 = Japanese male 1, JM2 = Japanese male 2.

There were large differences between speakers, as shown by the response distributions to the McGurk stimulus of each speaker for Finnish and Japanese participants (Figure 3). Consequently, it was important to account for such differences when analyzing cross-language differences.

To examine cross-language effects in the McGurk effect, we conducted logistic regression analyses with a GLMM for the three responses separately, with participants’ and speakers’ language (L1: FI or JP) as fixed factors and participants and speakers (eight different speakers) as random effects. Speakers as a random effect takes differences between speakers into account in the model. In reference to a previous study [29], we analyzed the proportion of responses (Pa, Ta, Ka) separately as binomial (0, 1) data.

For Pa responses, participants’ L1 did not have a significant effect (*β* = −0.534, *SE* = 0.394, *z* = −1.356, *p* = 0.175, *OR* = 0.586) but speakers’ L1 did (*β* = −2.85, *SE* = 0.248, *z* = −11.5, *p* < 0.001, *OR* = 0.058), as there were more Pa responses to FI than JP stimuli. Importantly, the interaction between participants’ and speakers’ L1 was significant (*β* = 0.506, *SE* = 0.087, *z* = 5.836, *p* < 0.001, *OR* = 1.659). The interaction indicated that the proportion of Pa responses for FI stimuli was higher in Finnish than Japanese participants.

For Ta responses, the effect of participants’ L1 was significant (*β* = −0.344, *SE* = 0.128, *z* = −2.694, *p* = 0.007, *OR* = 0.709) but speakers’ L1 was not (*β* = 0.556, *SE* = 0.583, *z* = 0.953, *p* = 0.341, *OR* = 1.743). Importantly, the interaction between participants’ and speakers’ L1 was significant (*β* = 0.506, *SE* = 0.046, *z* = 11.077, *p* < 0.001, *OR* = 1.658). The interaction indicated that the proportion of Ta responses for FI stimuli was higher in Finnish than Japanese participants.

For Ka responses, the effect of participants’ L1 was significant (*β* = −1.563, *SE* = 0.468, *z* = −3.338, *p* = 0.001, *OR* = 0.210) but speakers’ L1 was not (*β* = 1.088, *SE* = 0.628, *z* = 1.732, *p* = 0.083, *OR* = 2.969). Importantly, the interaction between participants’ and speakers’ L1 was significant (*β* = −1.290, *SE* = 0.050, *z* = −25.675, *p* < 0.001, *OR* = 0.275). The interaction indicated that the proportion of Ka responses for FI stimuli was lower in Finnish than Japanese participants, but opposite for JP stimuli.

### 3.2. The Audiovisual Congruent Stimuli

**Figure 4:**
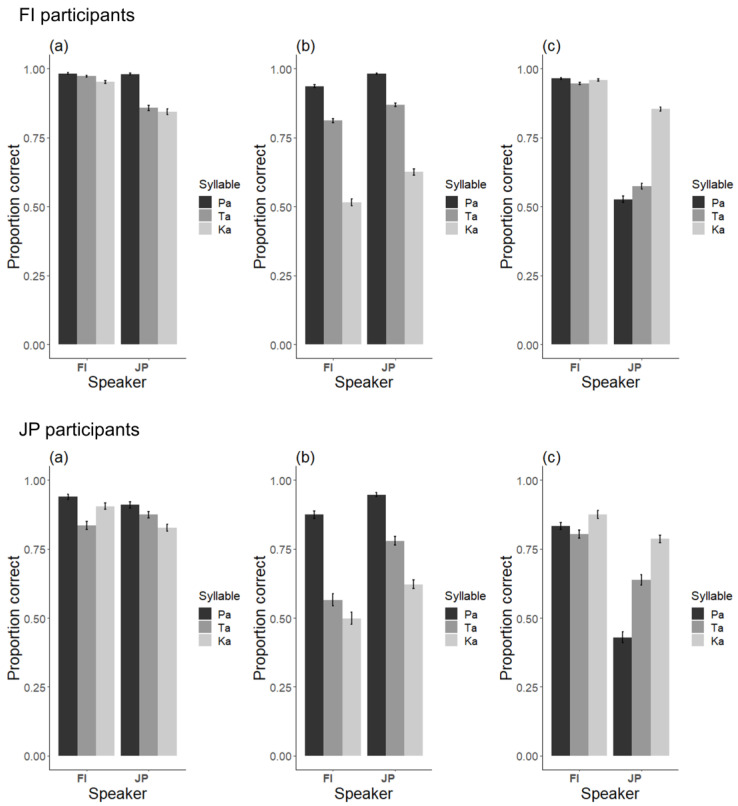
Proportion of correct responses to Finnish and Japanese stimuli in Finnish and Japanese participants: (**a**) audiovisual congruent stimuli; (**b**) visual stimuli; (**c**) auditory stimuli. The error bars represent the standard error of the mean.

The audiovisual congruent stimuli served as filler stimuli, providing exemplars of /pa/, /ta/ and /ka/ syllables among the McGurk stimuli. They are of little interest in the current study and are therefore treated very briefly. Figure 4a shows the proportion of correct responses for the three audiovisual congruent stimuli. We performed logistic regression analyses with GLMM to test the effects of language and syllable and found a significant interaction, indicating that for the JP stimuli, the proportion of correct responses was higher for /pa/ than for /ta/ (*β* = −1.57, *SE* = 0.27, *z* = 5.78, *p* < 0.001) and /ka/ (*β* = −1.13, *SE* = 0.26, *z* = 4.37, *p* < 0.001).

### 3.3. The Auditory and Visual Stimuli

Unisensory A/pa/ and V/ka/ were the key stimuli, since they were the components of the McGurk stimulus. Other unisensory stimuli served as fillers and are therefore treated briefly here. Figure 4b−c shows the mean proportion of correct responses for the visual and auditory stimuli, respectively. To examine the effect of speaker’s language for the unisensory stimuli, we performed logistic regression analyses with GLMM. 

For the auditory stimuli, the correct recognition rate was higher for FI than JP stimuli (*β* = −1.36, *SE* = 0.09, *z* = 15.11, *p* < 0.001). We also found that accuracy for /ka/ was higher than accuracies for /pa/ (*β* = −1.85, *SE* = 0.13, *z* = 14.52, *p* < 0.001) and /ta/ (*β* = −1.27, *SE* = 0.12, *z* = 10.83, *p* < 0.001) only for JP stimuli.

For the visual stimuli, the correct recognition rate was lower for FI than JP stimuli (*β* = 1.29, *SE* = 0.13, *z* = 9.77, *p* < 0.001). Also, the proportion of correct responses was higher for /pa/ than for /ta/ (*β* = −1.30, *SE* = 0.08, *z* = 17.08, *p* < 0.001) and /ka/ (*β* = −2.74, *SE* = 0.07, *z* = 37.87, *p* < 0.001) and higher for /ta/ than /ka/ (*β* = −1.44, *SE* = 0.05, *z* = 27.97, *p* < 0.001)) These findings applied to both FI and JP stimuli. 

**Figure 5:**
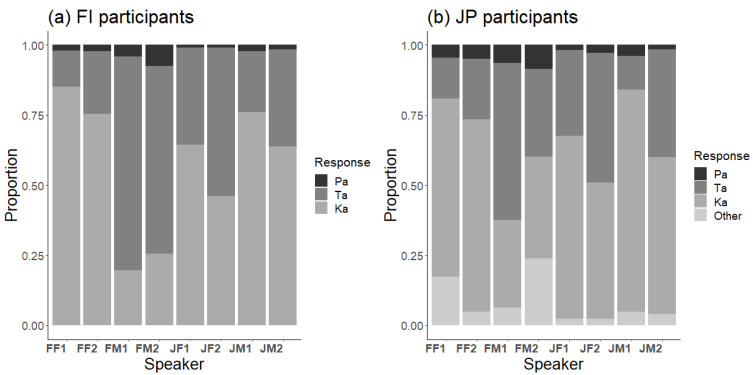
Response distributions to the visual /ka/ stimulus (V/ka/) for each of the eight speakers in (**a**) Finnish and (**b**) Japanese participants.

The response distributions to the visual /ka/ stimulus of each speaker for Finnish and Japanese participants showed large differences between speakers (Figure 5). Notably, V/ka/ was never perfectly recognized, and the most common incorrect responses were always Ta. The response patterns appeared to resemble those in the McGurk stimulus. Logistic regression analyses with GLMM with speakers as a fixed factor and participants as a random effect showed that speakers, participants and their interactions had significant effects. The FI and JP participant groups differed only in correct Ka responses (*β* = 1.31, *SE* = 0.20, *z* = 6.56, *p* < 0.001), with Finnish participants having a higher proportion of correct responses.

**Figure 6:**
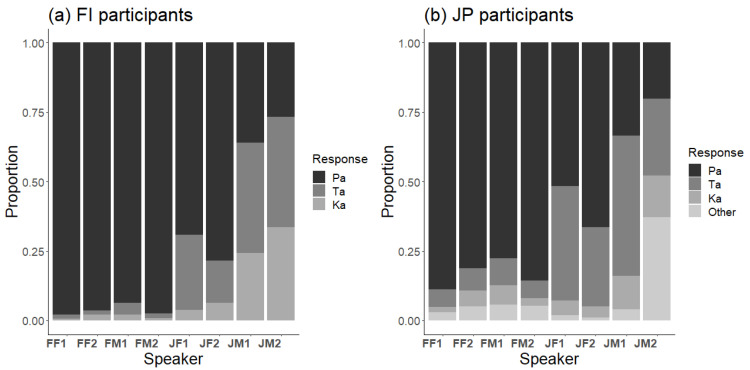
Response distributions to the auditory /pa/ stimulus (A/pa/) for each of the eight speakers in (**a**) Finnish and (**b**) Japanese participants.

Figure 6 shows the response distributions to the auditory /pa/ stimulus of each speaker for Finnish and Japanese participants. Again, there were differences between speakers. Logistic regression analyses with GLMM with speakers as a fixed factor and participants as a random effect showed that speakers, participants and their interactions had significant effects. The FI and JP participant groups differed in all responses (Pa: *β* = 1.78, *SE* = 0.30, *z* = 5.93, *p* < 0.001; Ta: *β* = −2.88, *SE* = 0.36, *z* = −4.69, *p* < 0.001; Ka: *β* = −1.04, *SE* = 0.51, *z* = −2.04, *p* = 0.04).

### 3.4. Correlations between Perception of the McGurk Stimulus and Unisensory Perception

**Figure 7:**
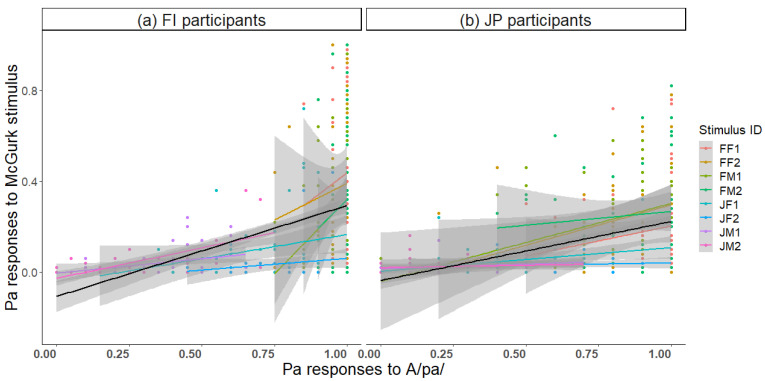
Correlation between correct Pa responses to the auditory /pa/ stimulus and Pa responses to the McGurk stimulus for each of the eight speakers and across speakers (black line) in (**a**) Finnish and (**b**) Japanese participants.

To investigate whether unisensory perception of the components A/pa/ and V/ka/ is reflected in audiovisual perception of the McGurk stimulus, correlation analyses were conducted. The proportion of correct Pa responses to unisensory A/pa/ correlated with Pa responses to the McGurk stimulus (Figure 7; *r* = 0.18, *p* < 0.001 FI participants; *r* = 0.19, *p* < 0.001 JP participants). Thus, the better the auditory recognition, the weaker the McGurk effect.

**Figure 8:**
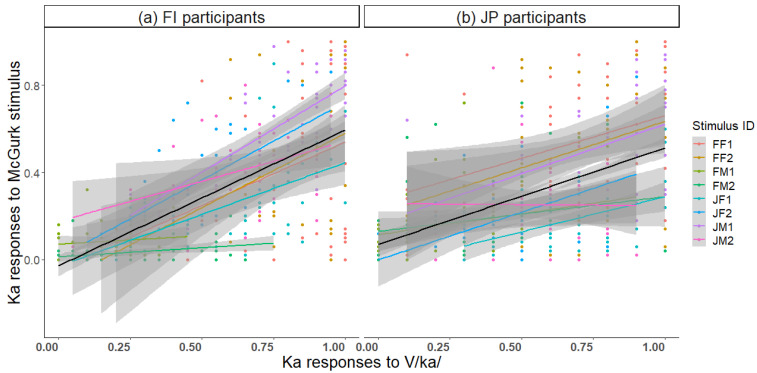
Correlation between response accuracy of the visual /ka/ stimulus and Ka responses to the McGurk stimulus for each of the eight speakers and across speakers (black line) in (**a**) Finnish and (**b**) Japanese participants.

**Figure 9:**
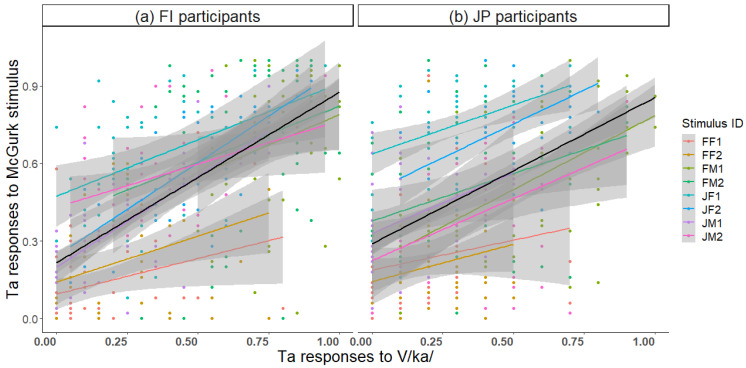
Correlation between Ta responses to visual /ka/ stimulus and Ta responses to the McGurk stimulus for each of the eight speakers and across speakers (black line) in (**a**) Finnish and (**b**) Japanese participants.

The proportion of correct Ka responses to unisensory V/ka/ correlated with Ka responses to the McGurk stimulus (Figure 8; *r* = 0.43, *p* < 0.001 FI participants; *r* = 0.30, *p* < 0.001 JP participants). Thus, the better the visual recognition, the stronger the visual influence on the McGurk effect; so, the McGurk stimulus was more often heard as Ka. The most frequent incorrect response to V/ka/ was Ta. There was a strong correlation between Ta responses to the McGurk stimulus and Ta responses to V/ka/ (Figure 9; *r* = 0.40, *p* < 0.001 FI participants; *r* = 0.38, *p* < 0.001 JP participants). Thus, the more frequently the visual /ka/ was lipread as /ta/, the more frequently the McGurk stimulus was heard as Ta.

## 4. Discussion

We studied cross-language effects in audiovisual speech perception between Finnish and Japanese speakers/stimuli and listeners/participants. The McGurk effect was used to measure visual influence on AV speech perception, i.e., the fewer the auditory responses, the stronger the McGurk effect. Finnish participants had a stronger McGurk effect when the speakers were Japanese rather than Finnish (Exp. 1). Taken alone, this would be consistent with the foreign language effect, showing stronger visual influence with stimuli produced by speakers of another language than one’s own L1 [15,16]. However, Japanese participants showed the same pattern, i.e., a stronger McGurk effect when the speakers were Japanese (Exp. 2). In turn, this finding alone would be consistent with an L1 advantage [17]. The only significant cross-language differences in the McGurk effect were that Finnish participants gave more Pa and Ta but fewer Ka responses to FI stimuli than Japanese participants. Regarding JP stimuli, there were more Ka responses by Finnish than Japanese participants. These findings suggest that visual influence may have been slightly stronger for foreign language stimuli but only for Finns. To emphasize L1 differences, GLMM with speakers as a random effect was used, but still, the cross-language effects were meagre. Finding rather similar patterns in listeners with different L1s is the most in line with the view that the McGurk effect does not primarily depend on the participants’ and speakers’ L1 but more on other factors, such as the properties of the stimuli that are used to assess the McGurk effect [18,30].

The auditory component of the McGurk stimulus, A/pa/, was accurately perceived for Finnish stimuli but poorly recognized for Japanese stimuli, even though the SNR was equal (0 dB) in all auditory stimuli. The acoustic cues may have been weaker in the original JP auditory stimuli; so, the noise had a more detrimental effect on their discrimination compared with FI auditory stimuli. Conversely, the visual component V/ka/ was more accurately perceived for Japanese than Finnish stimuli. According to the rule of inverse effectiveness [31,32], as well as maximum likelihood models of multisensory [33] and AV speech [34] perception, multisensory perception is more affected by the more reliable sensory modality. Here, auditory speech was relatively less reliable, and visual speech was more reliable for JP compared with FI stimuli. Consequently, visual influence was stronger with Japanese speakers. 

In a previous study, Ujiie and Takahashi (2022) found that Japanese listeners exhibited a stronger McGurk effect with Japanese than English speakers [17]. In that study, a preliminary experiment was conducted to prevent any differences in unisensory recognition accuracies between own and foreign language stimuli. This was because speaker familiarity differences were considered to be small and easily masked by other factors, such as the intelligibility of the auditory and visual components of the McGurk stimulus. Possible differences in unisensory perception may thus account for at least some of the variation in current and previous findings, since often unisensory perception, especially of visual speech, is not controlled for [18,20,22,35]. Unfortunately, Ujiie and Takahashi (2022) did not conduct the same experiment with English L1 participants [17]. In future studies, the languages to be compared should be used to provide both the stimuli and participants.

Cross-language effects were small, but the relationship between the McGurk effect and unisensory perception was rather strong, and differences between speakers appeared prominent. Response distributions to the McGurk stimulus (A/pa/V/ka/) showed notable differences between speakers, as did those to V/ka/ and to a lesser extent A/pa/ as well. For example, the proportion of Ta responses to the McGurk stimulus varied between 0.18 (FF1) and 0.69 (JF1) between speakers.

Generally, the visual—and to some extent auditory—response distribution was reflected in the response distribution to the McGurk stimulus. The more V/ka/ was confused with Ta, the more fusion responses there were to the McGurk stimulus. The better V/ka/ was correctly recognized, the more Ka responses there were to the McGurk stimulus. Also, the strength of the McGurk effect decreased as correct responses to its auditory component increased. These findings add to the previous ones showing that McGurk fusions are correlated with the participants’ ability to discriminate the visual place of articulation [26,27].

Surprisingly, few studies have addressed the role of individual speakers in eliciting the McGurk effect, even though the anecdotal practical experience of current authors (and presumably several others) is that when creating incongruent syllables, some individuals produce a very strong illusion; meanwhile, for others, the auditory syllable is mostly heard correctly. Jiang and Bernstein (2011) have conducted the most advanced analyses of A and V speech signals and their relationship to the perception of congruent and incongruent consonants to date [36]. Their methodology could be used to quantify differences between speakers.

Magnotti, Beauchamp and coworkers have pointed out that there are differences between McGurk stimuli but thus far have focused on fusion responses [24,30]. This focus is probably because fusion responses dominate in many experiments, while visual responses are often infrequent and thus not amenable to statistical analysis. Their causal inference of multisensory speech model explains how the McGurk effect arises from noisy sensory signals that are optimally combined, and it can be tested with different inputs, e.g., the effect of different speakers on audiovisual speech perception can be modeled [24]. (Also, other promising models exist [23,37].)

In the future, the contributions of stimulus properties, including individual differences between speakers, should be investigated in more depth. Both the acoustic and visual features of speech signals influence what is perceived. Modeling is the key to understanding how multisensory perception occurs. With regards to cross-language differences, there may be language-dependent cues in A and V speech, which should be taken into account when assessing potential differences in merging them when listening to one’s own or a foreign language. 

## 5. Conclusions

Cross-language influences on the McGurk effect were quite small for Finnish and Japanese speakers and listeners. However, differences between individual speakers were conspicuous, both in the McGurk effect and its auditory and visual components when presented alone. Unisensory perception was reflected in responses to the McGurk stimulus. These findings emphasize the need to investigate the stimulus features contributing to the McGurk effect and to use a large set of talkers as stimuli in cross-language and other studies on AV speech perception.

## Data Availability

The data presented in this study are available on request from the corresponding author.
